# Supplementary material for: Resolving the full spectrum of human genome variation using Linked-Reads
Source: Genome Res. 2019 Apr;29(4):635–45. doi: 10.1101/gr.234443.118 (PMC6442396; doi:10.1101/gr.234443.118)
Supplement: Supplemental Material [file supp_gr.234443.118_Supplemental_longranger-2.2.zip › longranger-2.2.tar.bz2!/lr2.2-public/lib/stan/cmdstan/stan/doc/api/html/classstan_1_1io_1_1writer-members.html]

Stan: Member List


|  |  |
| --- | --- |
|  | Stan  2.5.0  probability, sampling & optimization |


- Main Page
- Related Pages
- Namespaces
- Classes
- Files

- Class List
- Class Index
- Class Hierarchy
- Class Members

All Classes Namespaces Files Functions Variables Typedefs Enumerations Enumerator Friends Macros Pages

- stan
- io
- writer

stan::io::writer< T > Member List

This is the complete list of members for stan::io::writer< T >, including all inherited members.

|  |  |  |
| --- | --- | --- |
| array\_vec\_t typedef | stan::io::writer< T > |  |
| cholesky\_corr\_unconstrain(matrix\_t &y) | stan::io::writer< T > | inline |
| cholesky\_factor\_unconstrain(matrix\_t &y) | stan::io::writer< T > | inline |
| CONSTRAINT\_TOLERANCE | stan::io::writer< T > |  |
| corr\_matrix\_unconstrain(matrix\_t &y) | stan::io::writer< T > | inline |
| corr\_unconstrain(T &y) | stan::io::writer< T > | inline |
| cov\_matrix\_unconstrain(matrix\_t &y) | stan::io::writer< T > | inline |
| data\_i() | stan::io::writer< T > | inline |
| data\_r() | stan::io::writer< T > | inline |
| integer(int n) | stan::io::writer< T > | inline |
| matrix\_lb\_unconstrain(double lb, matrix\_t &y) | stan::io::writer< T > | inline |
| matrix\_lub\_unconstrain(double lb, double ub, matrix\_t &y) | stan::io::writer< T > | inline |
| matrix\_t typedef | stan::io::writer< T > |  |
| matrix\_ub\_unconstrain(double ub, matrix\_t &y) | stan::io::writer< T > | inline |
| matrix\_unconstrain(const matrix\_t &y) | stan::io::writer< T > | inline |
| ordered\_unconstrain(vector\_t &y) | stan::io::writer< T > | inline |
| positive\_ordered\_unconstrain(vector\_t &y) | stan::io::writer< T > | inline |
| prob\_unconstrain(T &y) | stan::io::writer< T > | inline |
| row\_vector\_lb\_unconstrain(double lb, row\_vector\_t &y) | stan::io::writer< T > | inline |
| row\_vector\_lub\_unconstrain(double lb, double ub, row\_vector\_t &y) | stan::io::writer< T > | inline |
| row\_vector\_t typedef | stan::io::writer< T > |  |
| row\_vector\_ub\_unconstrain(double ub, row\_vector\_t &y) | stan::io::writer< T > | inline |
| row\_vector\_unconstrain(const vector\_t &y) | stan::io::writer< T > | inline |
| scalar\_lb\_unconstrain(double lb, T &y) | stan::io::writer< T > | inline |
| scalar\_lub\_unconstrain(double lb, double ub, T &y) | stan::io::writer< T > | inline |
| scalar\_pos\_unconstrain(T &y) | stan::io::writer< T > | inline |
| scalar\_ub\_unconstrain(double ub, T &y) | stan::io::writer< T > | inline |
| scalar\_unconstrain(T &y) | stan::io::writer< T > | inline |
| simplex\_unconstrain(vector\_t &y) | stan::io::writer< T > | inline |
| unit\_vector\_unconstrain(vector\_t &y) | stan::io::writer< T > | inline |
| vector\_lb\_unconstrain(double lb, vector\_t &y) | stan::io::writer< T > | inline |
| vector\_lub\_unconstrain(double lb, double ub, vector\_t &y) | stan::io::writer< T > | inline |
| vector\_t typedef | stan::io::writer< T > |  |
| vector\_ub\_unconstrain(double ub, vector\_t &y) | stan::io::writer< T > | inline |
| vector\_unconstrain(const vector\_t &y) | stan::io::writer< T > | inline |
| writer(std::vector< T > &data\_r, std::vector< int > &data\_i) | stan::io::writer< T > | inline |
| ~writer() | stan::io::writer< T > | inline |


---

[ Stan Home Page ]

*© 2011–2014,
Stan Development Team.*
